# Attention-Based Temporal Encoding Network with Background-Independent Motion Mask for Action Recognition

**DOI:** 10.1155/2021/8890808

**Published:** 2021-03-27

**Authors:** Zhengkui Weng, Zhipeng Jin, Shuangxi Chen, Quanquan Shen, Xiangyang Ren, Wuzhao Li

**Affiliations:** ^1^Jiaxing Vocational and Technical College, Jiaxing, Zhejiang, China; ^2^Medical 3D Printing Center, The First Affiliated Hospital of Zhengzhou University, Zhengzhou, Henan, China; ^3^School of Electrical Engineering, Zhengzhou University, Zhengzhou, Henan, China; ^4^Wenzhou Polytechnic, Wenzhou, Zhejiang, China

## Abstract

Convolutional neural network (CNN) has been leaping forward in recent years. However, the high dimensionality, rich human dynamic characteristics, and various kinds of background interference increase difficulty for traditional CNNs in capturing complicated motion data in videos. A novel framework named the attention-based temporal encoding network (ATEN) with background-independent motion mask (BIMM) is proposed to achieve video action recognition here. Initially, we introduce one motion segmenting approach on the basis of boundary prior by associating with the minimal geodesic distance inside a weighted graph that is not directed. Then, we propose one dynamic contrast segmenting strategic procedure for segmenting the object that moves within complicated environments. Subsequently, we build the BIMM for enhancing the object that moves based on the suppression of the not relevant background inside the respective frame. Furthermore, we design one long-range attention system inside ATEN, capable of effectively remedying the dependency of sophisticated actions that are not periodic in a long term based on the more automatic focus on the semantical vital frames other than the equal process for overall sampled frames. For this reason, the attention mechanism is capable of suppressing the temporal redundancy and highlighting the discriminative frames. Lastly, the framework is assessed by using HMDB51 and UCF101 datasets. As revealed from the experimentally achieved results, our ATEN with BIMM gains 94.5% and 70.6% accuracy, respectively, which outperforms a number of existing methods on both datasets.

## 1. Introduction

Understanding and the process to recognize actions of humans in realistic videos remains a challenge due to the diversified objects, ambiguous human motions, and complex relationship between scenes and human beings. From traditional handcraft features [1] to deep learning-based approaches [2], impressive progress has been made owing to the development of powerful pattern recognition approaches over the past few years. Meanwhile, the convolutional neural network (CNN) has become popular for human action recognition [3–5]. However, compared with the progress in still image classification [6], CNN action recognition's performance improvements is relatively small.

**Figure 1:**
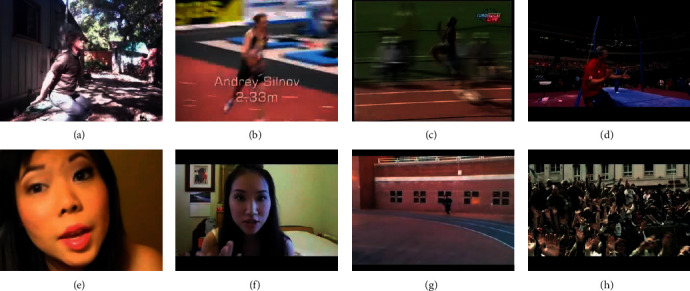
Some sampled video frames and their corresponding categories. (a) BodyWeightSquats, (b) HighJump, (c) LongJump, (d) StillRings, (e) ApplyEyeMakeup, (f) ApplyLipStick, (g) CricketBowling, and (h) CliffDiving.

Different with the still image classifying, a special property for action recognition refers to scattered background and dynamic perspective. According to Figure 1, some example frames with poor conditions are illustrated in the first row. Thus, some existing video action recognition methods seek the diminishment of the interfere of different visual appearances based on the detection of interest points of space and time [7], creating trajectories of motion [8], or splitting the object that moves [9] prior to their feeding to CNNs. Nevertheless, the mentioned approaches have two major drawbacks. On the one hand, several subtle motions carrying vital data are likely to be lost under the extremely sensitive threshold. On the other hand, discriminative characteristics are hard to extract from the taken areas with the relationship to action since the mentioned foreground areas are likely to show a spatial coherence [10].

In addition, the other distinct feature exhibited by the video refers to temporal dimension. It is difficult for 3D-CNN architectures [11] to obtain higher performance when compared with the sophisticate improved dense trajectory (IDT) [12] at the early stage because only a few frames rather than the whole video may be covered by comparatively small 3D convolution. More recently, researchers focus on combing the CNNs with RNNs such as LSTM [13] to model the temporal configuration within the video. The other one focuses on aggregating the frame-level descriptors through different feature encoding approaches (e.g., the average or maximal pooling along temporal dimension [14], vector of descriptors with local aggregation (VLAD) [15], and temporal pyramid pooling (TPP) [16]).

Nevertheless, in the absence of semantic data guiding at a high level, the representation of actions under the extraction with the use of the mentioned methods fail to have the expression of the frames with the higher relevance inside a video in a selective manner; accordingly, the residual noise is likely to increase unavoidably based on frames with any relevance [17]. The second row of Figure 1 gives some sampled video frames and their corresponding categories that are inappropriate for classification.

**Figure 2:**
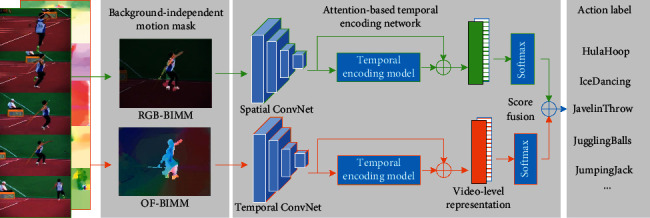
The framework of the temporal encoding network on the basis of attention here with background-independent motion mask.

In view of the above observations and analysis, a novel framework is developed, termed as temporal encoding network (ATEN) on the basis of attention with background-independent motion mask (BIMM) for video action recognition (Figure 2). Inspired by the successful application of saliency technology in still images [18, 19], one motion object segmentation approach on the basis of boundary prior receives the introduction through the combination of the minimal geodetic distance based on dynamic contrast segmenting strategic procedure in an undirected weighted graph. Subsequently, we build the BIMM for enhancing the object that moves based on the suppression of the background motion with relevance within the respective frame. The attention mechanism in ATEN does not process all sampled frames equally but automatically highlight the semantic vital frames.

The major contribution here is elucidated as follows:

- We develop an overall automatic background-independent motion mask (BIMM) for enhancing the moving objects based on sophisticated background by complying with one emerging motion segmenting approach on the basis of boundary prior
- An attention-based temporal encoding network (ATEN) is developed for video action recognition. Instead of solving the features at the frame level in an equal manner, one uncomplicated and significant attention mechanism based on long ranges is proposed
- We conduct extensive experimental processes on two difficult datasets for demonstrating that the framework here statistically outclasses a number of existing methods by average 7.8% and 4.0%

## 2. Related Work

### 2.1. Approaches on the Basis of Handcraft Features

Prior to the extensive CNN application, methods under the handcrafting process are dominant in action-recognizing field. For instance, local spatiotemporal descriptors with the fisher vector (FV) [20] and classical bag of words (BoW) [21] model had shown good recognition performance. Descriptors on the basis of histogram under the calculation following the trajectory [12, 22] achieved high-prospect outcomes in human action-recognizing task. In [22], histograms of optical flow (HOF) and oriented gradients (HOG) received the extraction in the respective spatial-temporal interest point to model local motion information. Wang et al. [22] conducted the extraction of dense trajectory based on the track of dense point frame by frame under a range of scales. Four descriptors, namely, HOG, HOF, motion boundary histogram (MBH) [22], and trajectory shape (TS) [22] received the extraction along every dense trajectory to represent the action. To eliminate the impact of camera motion, an improved dense trajectory (IDT) was proposed by using a human detector to extract trajectory more concentrated in the action-related areas [12]. In order to capture motion features more effectively, as suggested by Vig et al. [23], the recognition performance is able to be kept with just 30–40% descriptors chosen from dense trajectories. In this end, Yi and Lin [24] and Xu et al. [25] aimed to select dense trajectories from salient motion areas and encoded them into a compact video representation. Unlike [24, 25] utilizing video saliency to select action-related trajectory, the authors in [26] proposed a length-variable edge trajectory extracted from edge points to model different speeds of motion. These approaches commonly focused on capturing edge, corner, and motion features through trajectories and were successful in recognizing relatively simple actions. However, it remains challenging to recognize various long-term complex human actions.

### 2.2. Approaches on the Basis of Deep Learning

Progresses for deep learning over the past few years lead to impressive applications for video action recognition. To be specific, pretrained CNN transfer learning with limited training information shows great performance in [27–30]. For instance, Simonyan and Zisserman [27] pretrained a 2-stream CNN composed of spatial CNN and temporal CNN using the ImageNet dataset to capture features from single static frame and motions, respectively. Tran et al. [28] extended the 3D-CNN [11] by learning spatiotemporal features from adjacent frames with 3D convolution and pooling. Sun et al. [4] introduced one emerging spatiotemporal CNN structure which factorized 3D convolution into 2D spatial convolution to reduce the computation complexity. In [29], a new three-stream CNN model was proposed, which is derived from two-stream CNN. Wang et al. [30] proposed a hybrid representation which combined the merits from both IDT [12] and two-stream CNN to form the trajectory-pooled deep convolution descriptor (TDD). Feichtenhofer et al. [31] discovered a deficiency in two-stream CNN and further improved it with convolutional fusion of two streams. Besides, the selection of informative areas to extract features refers to a reasonable method for promoting action recognition performance. Cheron et al. [14] proposed one CNN (P-CNN) on the basis of pose which was different from conventional two-stream architecture. In [14], the human pose was first detected and tracked over time, and then the CNN features were obtained separately from each part of body in each frame. However, the performance of P-CNN remains unsatisfied because pose estimation in natural images remains challenging. In [10], a fast and accurate video representation method based on salient areas of motion was proposed, which can extract the most useful features for classification. Rui et al. [32] developed one tube CNN (T-CNN) based on 3D convolution operation to recognize actions. However, most CNN-based methods are subject to false recognition and detail missing, since merely coarse global features at the frame level are adopted [3]. Moreover, the other limitation of the aforementioned CNN frameworks is that they usually focus more on capturing appearance and short-term features rather than multistaged temporal information. Considering that the temporal information is critical for video classification, Shi et al. [33] proposed an effective representation for long-term motions by extending two-stream framework with an informative representation named sequential trajectory texture (STT). Wang et al. [34] developed one video-level framework termed as the temporal segmenting network (TSN), aiming to utilize long-term temporal information by a sparse sampling strategy. Meanwhile, several works [13, 35, 36] introduced recurrent neural networks (RNNs) such as LSTM, which can preserve the previous states in memory cells for modelling the data in video in a long term. Donahue et al. [13] designed a unified model called LRCN which connected LSTM with CNNs to describe actions. A hierarchical LSTM network was proposed in [36], which enables to incorporate the information from three aspects, namely, long-term temporal structure, short-term motion, and static spatial in terms of complex action classification.

Lately, applying models of attention arouses rising attention inside a range of aspects (e.g., image captioning [37] and machine translation [38]). Several methods on the basis of attention were designed to recognize actions. Li et al. [39] employed a dual attention ConvNet (DANet) to deal with the computational cost of two-stream framework. Li et al. [40] developed one end-to-end structure based on the adaptive spatiotemporal attention model. Liu et al. [41] proposed one unified spatiotemporal attention network (STAN) to recognize video actions, one general attention neural cell called AttCell was devised to estimate the attention probability in the respective space site and for the respective video segment inside one temporal sequence. Yang et al. [42] developed a spatial-temporal attentive convolutional neural network (STA-CNN), incorporating one spatial attention system and one temporal attention system inside one convolutional network under the unification for recognizing video action. However, majority of the presented attention models to recognize actions employ RNN structures for implementing attention systems. The main defect exhibited by RNN refers to its instable property within training. The ATEN here receives the implementation with no use of RNN. As opposed to the mentioned, neural network structure is adopted for building the temporal encoding module.

## 3. Background-Independent Motion Mask

### 3.1. Overview of the Boundary Prior-Based Motion Object Segmentation for BIMM

**Figure 3:**
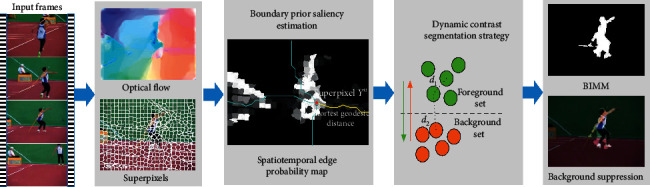
Overview of boundary prior-based motion segmentation for BIMM.

Figure 3 overviews the boundary prior-based motion segmentation for BIMM. Initially, the respective input frame receives the segmenting process to superpixels. One weighted graph that is not directed, with the respective superpixel recognized to be a node, is built inside the respective frame under the segmentation, and the nearby relationship of superpixels is presented by the edge between two nodes. Inspired by [19, 43], we define the geodesic distance as the cumulative weighted shortest path between two superpixels in the constructed graph. According to observation, the objects that move are constantly surrounded by the superpixels exhibiting great edge spatiotemporal value and the saliency probability receives the computation to be the minimal geodesic distance to the respective superpixel to the frame boundary. For extracting appearance and motion data in the area with the relationship to action, 2 kinds of the edge received the extraction from the frame: motion edges under the estimation based on optical flow via 2 consecutive frames and static apparent edges in the single static frame. Then, one adaptive threshold receives the design for generating the coarse saliency map based on the process to split the superpixels to foreground superpixel set as well as background superpixel set. Moreover, one dynamic contrast segmenting strategic procedure receives the introduction for gaining the precise motion areas based on the computation of the geodesic distance of each 2 superpixels of foreground and background sets inside the graph. Foreground superpixels exhibiting greater mean geodesic distance to the background superpixels are considered to be pseudoforeground superpixels and introduced to the background set and vice versa. Lastly, the respective frame background receives the suppression based on halving of the brightness of pixels not within the precise motion area.

### 3.2. Spatiotemporal Edge Generation

Image preprocessing [44, 45] also plays an important role in BIMM. Given a frame sequence *F* = {*F*^1^, *F*^2^,…, *F*^*k*^}, *P*_*i*_^*k*^=(*x*_*i*_^*k*^, *y*_*i*_^*k*^) denotes the *i*-th pixel in frame *F*^*k*^. We calculate the apparent edge probability map *E*_static_^*k*^(*P*_*i*_^*k*^)=*E*_static_^*k*^(*x*_*i*_^*k*^, *y*_*i*_^*k*^) corresponding to *k*-th frame *F*^*k*^ and pixel *P*_*i*_^*k*^ using Canny edge detector [46]. Let *ω*_*i*_^*k*^=(*u*_*i*_^*k*^, *v*_*i*_^*k*^) be the optical flow of *i*-th pixel in frame *F*^*k*^, where *u* and *v* are the horizontal and vertical component, respectively, then we calculate the motion gradient *E*_motion_^*k*^(*ω*_*i*_^*k*^) of the optical flow [47] *ω*_*i*_^*k*^ as

(1) $$\begin{array}{c} E_{\text{motion}}^{k}\left(\omega_{i}^{k}\right)=\left\|\nabla \omega_{i}^{k}\right\|=\left\|\frac{\partial \omega_{i}^{k}}{\partial u_{i}^{k}}+\frac{\partial \omega_{i}^{k}}{\partial v_{i}^{k}}\right\|. \end{array}$$

Subsequently, based on SLIC, the present study segments the frame to superpixels [48]. To be specific, the color space of the image is transformed from RGB to LAB firstly, and a five-dimensional feature vector is composed of three components from LAB and the distance between two pixels *x*, *y*. Then, the K-means algorithm is used to cluster the pixels according to the certain distance measurement. Finally, the superpixels with relatively uniform size are generated.*Y*^*k*^={*Y*_1_^*k*^, *Y*_2_^*k*^,…, *Y*_*n*_^*k*^} is denoted as the superpixel set in frame *F*^*k*^. Based on the static edge map *E*_static_^*k*^, we calculate the superpixel *Y*_*n*_^*k*^ edge probability to be the pixels on average with ten maximal edge probability values in *Y*_*n*_^*k*^. Likewise, the gradient magnitude of motion is obtained based on *E*_motion_^*k*^. Through the mentioned processes, two superpixel-based edge probability maps *E*_static_^*k*^ and *E*_motion_^*k*^ are developed. Next, one spatiotemporal edge probability map *E*^*k*^(*Y*^*k*^) is able to be developed [19] to be the element-wise multiplying of *E*_static_^*k*^ and *E*_motion_^*k*^:

(2) $$\begin{array}{c} E^{k}\left(Y^{k}\right)=E_{\text{static}}^{k}\circ E_{\text{motion}}^{k}. \end{array}$$

The reason for computing *E*^*k*^(*Y*^*k*^) is that both static and motion information capable of providing useful information for motion object segmentation is considered. If the foreground motion region is different from the background, optical flow gradient magnitude is required to achieve high value close to the foreground region compared with background. Moreover, the static edge probability provides one instructing element in terms of the apparent boundaries by complying with the appearance in the RGB frame. When both kinds of information fused using (2), the final spatiotemporal edge probability map *E*^*k*^(*Y*^*k*^) is able to point out the location of the foreground moving object.

### 3.3. Boundary Prior Saliency Estimation

In terms of the respective frame *F*^*k*^, one weighted graph that is not directed is built to be *g*^*k*^={*Y*^*k*^, *e*^*k*^} with superpixels *Y*^*k*^ as nodes and the edges between pairs of adjacent superpixels as *e*^*k*^. Under the built configuration, one |*Y*^*k*^| × |*Y*^*k*^| adjacency matrix of the graph *W*^*k*^(*m*, *n*) receives the development, with the definition of the element-wise multiplying of *e*^*k*^(*m*, *n*) and *w*_*mn*_^*k*^, where *e*^*k*^(*m*, *n*) represents the edge between adjacent superpixels *Y*_*m*_^*k*^ and *Y*_*n*_^*k*^, if there is an edge between two superpixels, and *e*^*k*^(*m*, *n*) is 1; otherwise, *e*^*k*^(*m*, *n*) is 0. *w*_*mn*_^*k*^ represents the weight of adjacent superpixels *Y*_*m*_^*k*^ and *Y*_*n*_^*k*^:

(3) $$\begin{array}{c} W^{k}\left(m,n\right)=e^{k}\left(m,n\right)\circ w_{mn}^{k},\\ w_{mn}^{k}=\left\|E^{k}\left(Y_{m}^{k}\right)-E^{k}\left(Y_{n}^{k}\right)\right\|, \end{array}$$

where *E*^*k*^(*Y*_*m*_^*k*^) and *E*^*k*^(*Y*_*n*_^*k*^) express the spatiotemporal boundary probability of superpixels *Y*_*m*_^*k*^ and *Y*_*n*_^*k*^, separately. For emphasizing the foreground motion objects with large spatiotemporal edge data or having the surrounding by areas with high spatiotemporal edge data, the present study adopts geodesic distance for computing one coarse object probability map. The geodesic distance *d*_*g*_(*Y*_*m*_^*k*^, *Y*_*n*_^*k*^, *g*^*k*^) in graph *g*^*k*^ has the definition of the cumulative weighted shortest path over all possible paths between *Y*_*m*_^*k*^ and *Y*_*n*_^*k*^:

(4) $$\begin{array}{c} d_{g}\left(Y_{m}^{k},Y_{n}^{k},g^{k}\right)=\min\left\{\sum_{Y_{m}^{k}}^{Y_{n}^{k}}\left|W^{k}\left(m,n\right)\cdot e^{k}\left(m,n\right)\right|\right\}. \end{array}$$

By complying with the definition of geodesic distance *d*_*g*_, we can find that supposing a superpixel is located externally from the moving object, there possibly exists a path to the frame boundaries not passing through any superpixels which have high spatiotemporal edge value. For this reason, the geodesic distance of such superpixel is relatively small. In contrast, supposing a superpixel is inside the moving object, the superpixel should have the surrounding by the superpixels exhibiting large spatiotemporal edge value, increasing the geodesic distance to the frame boundaries. When the superpixel is on the edge of the moving object, the path to one of the boundaries does not pass through the moving object (except itself) and the geodesic distance of the mentioned superpixel turns out to be comparatively low. However, our graph is very sparse, and although the superpixel is on the edge of the moving target, its own spatiotemporal edge value is much larger than those of the background superpixels. Likewise, the boundary prior saliency value *S*_*n*_^*k*^ in terms of the respective superpixel *Y*_*n*_^*k*^ is computed by

(5) $$\begin{array}{c} S_{n}^{k}=\underset{q\in Q^{k}}{\min}\left\{d_{g}\left(Y_{n}^{k},q,g^{k}\right)\right\}, \end{array}$$

where *Q*^*k*^ denotes the superpixels from four boundaries in each frame *F*^*k*^. All saliency values in *S*_*n*_^*k*^ are normalized to [0, 1]. Under the saliency value *S*_*n*_^*k*^, the coarse saliency map can be generated based on a self-adaptive threshold, dividing the superpixels into background set ${\hat{B}}^{k}$ and foreground set ${\hat{O}}^{k}$. The self-adaptive threshold *θ*^*k*^ for each frame *F*^*k*^ is computed by

(6) $$\begin{array}{c} \theta^{k}=\mu \left(S_{n}^{k}\right)=\frac{1}{n}\sum_{i\in n}S_{i}^{k}, \end{array}$$

where *μ*(·) denotes the mean value of all superpixels within frame *F*^*k*^ by saliency value *S*_*n*_^*k*^. Subsequently, the superpixels in the respective frame *F*^*k*^ can be cataloged to background and foreground set:

(7) $$\begin{array}{c} {\hat{O}}^{k}=\left\{Y_{n}^{k}|S_{n}^{k}>\theta^{k}\right\},\\ {\hat{B}}^{k}=Y^{k}-{\hat{O}}^{k}. \end{array}$$

For obtaining the refined motion object region, we add one dynamic contrast segmentation strategy by comparing both inter- and intrageodesic distance between foreground and background sets. Three kinds of distances, namely, *d*_*OO*_, *d*_*OB*_, and *d*_*BB*_ are utilized as the measurement for the superpixels $Y^{k}={\hat{O}}^{k}\cup {\hat{B}}^{k}$ in each frame *F*^*k*^:

(8) $$\begin{array}{c} d_{\text{OO}}=\mu \left(\underset{Y_{\text{n}}^{k},Y_{n+1}^{k}\in {\hat{O}}^{k}}{\sum }\left[d_{g}\left(Y_{n}^{k},Y_{n+1}^{k},g^{k}\right)\right]\right),\\ d_{\text{OB}}=\mu \left(\underset{Y_{n}^{k}\in {\hat{O}}^{k},Y_{n+1}^{k}\in {\hat{B}}^{k}}{\sum }\left[d_{g}\left(Y_{n}^{k},Y_{n+1}^{k},g^{k}\right)\right]\right),\\ d_{\text{BB}}=\mu \left(\underset{Y_{n}^{k},Y_{n+1}^{k}\in {\hat{B}}^{k}}{\sum }\left[d_{g}\left(Y_{n}^{k},Y_{n+1}^{k},g^{k}\right)\right]\right), \end{array}$$

where *d*_OO_ denotes the mean geodesic distance between two foreground superpixels. The higher the geodesic distance value, the closer the relationship between the superpixels. Similarly, *d*_OB_ and *d*_BB_ are also adopted as the measurement to describe inter- and intrarelationship among superpixels in one frame. Thus, we define the refined motion object areas *O*^*k*^ of *k*-th frame as

(9) $$\begin{array}{c} O^{k}=\left\{Y^{k}\in {\hat{O}}^{k}|d_{\text{OO}}>d_{\text{OB}}\right\}\cup \left\{Y^{k}\in {\hat{B}}^{k}|d_{\text{BB}}\leq d_{\text{OB}}\right\},\\ B^{k}=Y^{k}-O^{k}. \end{array}$$

**Figure 4:**
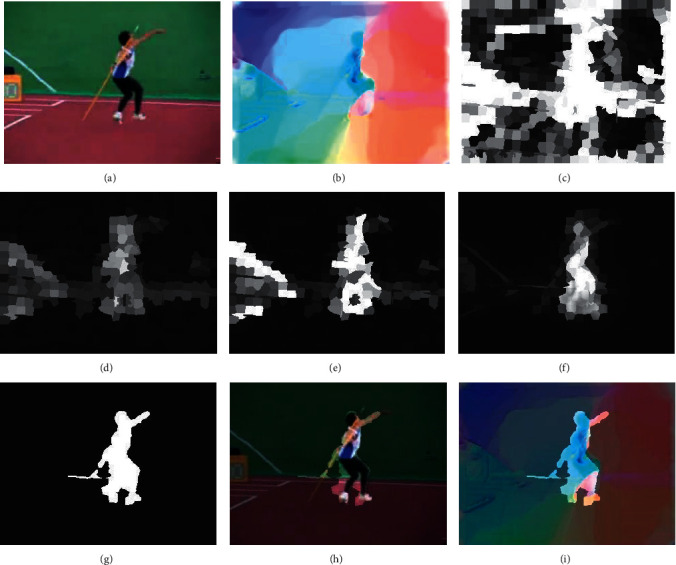
Visualization of boundary prior-based motion object segmentation for BIMM. (a) Source frame *F*_*k*_. (b) Optical flow *ω*^*k*^ of *F*_*k*_. (c) Superpixel-based optical flow gradient magnitude *E*_motion_^*k*^. (d) Superpixel-based static edge map *E*_static_^*k*^. (e) Spatiotemporal edge probability map *E*_*k*_. (f). Boundary prior saliency map *S*_*n*_^*k*^ for each superpixel *Y*_*n*_^*k*^ using geodesic distance to the four frame boundaries (g) BIMM generation from refined motion object areas by using dynamic contrast segmentation strategy. (h) Source frame *F*_*k*_ is decomposed into background set *B*^*k*^ and foreground set *O*^*k*^ after background suppression using BIMM, and the bright areas indicate the refined motion object areas. (i) The optical flow *ω*_*i*_^*k*^ of each frame *F*_*k*_ is treated in the same way to form OF-BIMM.

The main rationale behind (9) is both inter- and intradifferences between two superpixel sets are taken into consideration in the proposed dynamic contrast segmentation strategy. The intermediate results of BIMM are shown in Figure 4.

### 3.4. Background Suppression

By complying with the classified superpixels *Y*^*k*^ in each frame *F*^*k*^, the corresponding BIMM is computed by binarizing the values *S*_*n*_^*k*^ in the coarse saliency map. If *S*_*n*_^*k*^(*i*, *j*) ∈ *B*^*k*^, then *S*_*n*_^*k*^(*i*, *j*)=0; otherwise, *S*_*n*_^*k*^(*i*, *j*)=1. Next, we suppress the background pixels in *F*^*k*^ to improve the importance of foreground motion objects. We first transform the *F*^*k*^ in RGB color space to HSI color space, halving the I component of background areas where *S*_*n*_^*k*^(*i*, *j*)=0, and then do an opposite operation to reverse the frame back to RGB color space. As a result, a BIMM-based frame can be obtained and denoted as RGB-BIMM. Similarly, to construct the OF-BIMM, the optical flow *ω*_*i*_^*k*^ of each frame *F*^*k*^ is treated in the same way.

## 4. Video-Based Action Recognition Using ATEN

### 4.1. Temporal Structure Modelling

For each video *V*, we divide it into *N* segments along the temporal domain, i.e., *V*={*S*_*k*_}_*k*=1_^*N*^, where *S*_*k*_ is the *k*-th segment. Thus, a sequence of video snippets *T*_1_, *T*_2_, *T*_3_,…, *T*_*N*_ is obtained by randomly sampling each frame from the corresponding segment *S*_*N*_. Then, the temporal structure of a video *V* can be modelled as follows:

(10) $$\begin{array}{c} \text{TS}\left(V\right)=C\left(ℱ\left(T_{1};W\right),ℱ\left(T_{2};W\right),\ldots ,ℱ\left(T_{N};W\right)\right), \end{array}$$

where *ℱ*(*T*_*N*_; *W*) is the function of a CNN with parameter W which represents the convolution results of snippet *T*_*N*_. Here, *T*_*N*_={*T*_*N*_^RGB−BIMM^, *T*_*N*_^OF−BIMM^}, which represents the two modalities RGB-BIMM and OF-BIMM from the *N*-th snippet. *C* is the snippet-level aggregation function, which aggregates all the features learned from the base model via a temporal attention model to obtain a video-level representation.

### 4.2. Attention-Based Temporal Encoding Network

**Figure 5:**
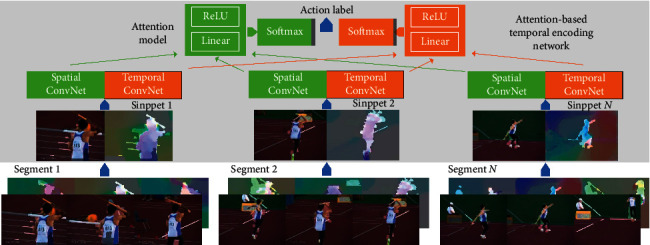
The overall framework of the attention-based temporal encoding network.

We do not use the single RGB frame or optical flow stacks in our framework, and the proposed ATEN works through the snippets that receive the sampling process in a sparse manner from the overall video. The respective snippet in the sequence generates its own feature representation. The overall framework of the attention-based temporal encoding network is shown in Figure 5. We use *ℱ*(*T*_*N*_; *W*) to represent any given one from the above two modalities (RGB-BIMM and OF-BIMM). A soft attention *e*_*i*_, *i* *=* 1,…, *N* is computed for each segment through one multiple-layer perceptron in the output with the full connection, which is defined as

(11) watt=ReLUWattℱTi;W+batt,ei=fattwatt,ℱTi;W=wattTℱTi;W,wi=expei∑j=1Nexpej,

where *w*_att_, *W*_att_, and *b*_att_ are the parameters of the attention model and the soft attention coefficient *e*_*i*_ in each *w*_att_ is then normalized to guarantee the sum of *e*_*i*_ is 1. The obtained temporal attention weight *w*_*i*_ is used to characterize the importance of the *i*-th snippet. Specifically, a degenerate case appears if *w*_*i*_=1/*N*, i.e., all snippets have equal importance.

Therefore, the segment-level aggregation function *C* is formed as *C*=∑_*i*=1_^*N*^*w*_*i*_*ℱ*(*i*_1_; *W*) to obtain a video-level video representation. Finally, the output of the ATEN is modelled as follows:

(12) $$\begin{array}{c} \text{ATEN}\left(T_{1},T_{2},T_{3},\ldots ,T_{N}\right)=ℋ\left(C\left(ℱ\left(T_{1};W\right),ℱ\left(T_{2};W\right),\ldots ,ℱ\left(T_{N};W\right)\right)\right). \end{array}$$

*C* combines the output from each snippet to obtain a fix-length feature vector, and based on this vector, *ℋ* aims to predict the probability score of the respective class in terms of the videos. Accordingly, the common multiclass activation function softmax is taken for *ℋ*. Based on the integration with the set softmax loss, we define the eventual loss function according to the aggregation function *C* as

(13) $$\begin{array}{c} ℒ\left(y,C\right)=-\sum_{i=1}^{K}y_{i}\log\left(\frac{\exp\left(C_{i}\right)}{\sum_{j=1}^{K}\exp\left(C_{j}\right)}\right), \end{array}$$

where *K* is the number of action categories and *y*_*i*_ is the action label corresponding to category *i*. It is worth mentioning that the gradients of the loss function can freely backpropagate, and the whole framework can be trained in an end-to-end manner because the entire ATEN is differentiable with the attention model directly embedded.

## 5. Experiments

### 5.1. Datasets and Evaluation Protocol

The HMDB51 [49] dataset is collected from variety of sources. There are a total of 6766 videos distributed in 51 action categories, each containing a minimum of 100 clips. We follow the original protocol using the first train-test splits and report the accuracy.

The UCF101 [50] dataset consists of 13320 videos falling into 101 action categories. UCF101 provides the largest diversity in terms of actions and the videos from the same group may share similar background and viewpoint. We evaluate our framework on the first train-test splits and report the accuracy.

### 5.2. Implementation Details

At the data preprocessing phase, the TVL1 optical flow algorithm [47] is employed. Specifically, the optical flow receives the discretization to [0, 255] for ensuring the information consistent property with RGB frames. Subsequently, the proposed BIMM is achieved by using both datasets for generating RGB-BIMM and OF-BIMM as the input of ATEN. Similar to [34], the temporal segment number is set to 6 empirically to guarantee the stability of the whole framework. In ATEN, we choose the public available Inception with Batch Normalization (BN-Inception) network as the base network here from [51] for its high efficient and accurate property. At the training stage, the mini-batch SGD optimizer is employed by setting batch size to 128 and setting momentum to 0.9. Specific to spatial ATEN, the network is initialized based on pretrained models from ImageNet [52] and the learning rates is first set to 0.001 and subsequently reduces to its 1/10 after per 1500 iterations. The iteration finally stopped at 3500. Specific to temporal ATEN, the cross-modality pretraining strategy proposed in [34] is employed based on the exploitation of the learned spatial models for initializing the temporal stream. The learning rate is set to 0.005, downregulated by a factor of 10 after 12K and 16K iterations. The max iteration is set to 18K. For data augmentation, we utilize the scale jittering and random horizontal flipping for reducing the risk of overfitting as advised in [34]. During the testing phase, we select 24 RGB-BIMM or OF-BIMM stacks from the action frames and then divide them into six snippets with each has four sampled frames according to temporal segments *N* = 6. Meanwhile, we crop 4 corners, 1 centre, and horizontal flipping of them from the sampled frames for assessing the ATEN. Finally, more credits are given to the temporal stream by setting its weight to 1.5 and that of the spatial stream to 1 for the fusion of two streams.

### 5.3. Evaluation on HMDB51

**Figure 6:**
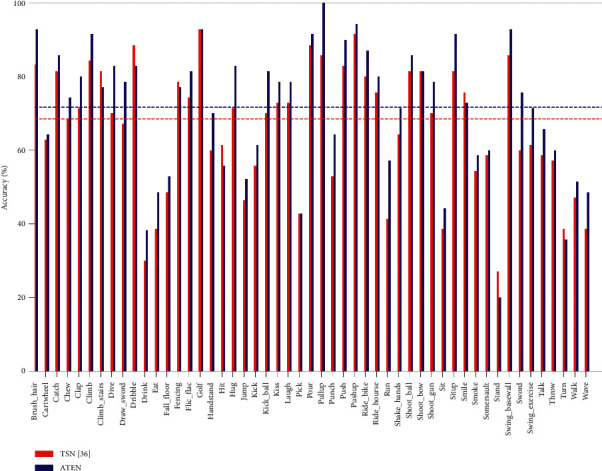
Performance comparison between ATEN and TSN (two modalities) [34] on HMDB51.

Here, the effect exerted by ATEN with BIMM on HMDB51 is shown in Table 1. We set TSN [34] as the baseline architecture in this section owing to its good performance. The performance of TSN here is implemented according to the reported settings (two modalities). We gain 59.7%, 62.2%, and 70.6% on spatial, temporal, and two-stream ATEN, respectively. The improvement of our ATEN (two modalities) over TSN is 2.1% on HMDB51 dataset which verifies the importance of modelling long-term temporal information. Furthermore, this study explores the improvement of performance of our ATEN over TSN on individual category.  Table 2 lists the top-10 action classes with the biggest increment on HMDB51. It is not difficult to find that several action categories with the highest performance increment are easy-confused actions, such as “Run,” “Pullup,” and “Dive,” demonstrating the proposed framework to be effective as well. Moreover, the proposed ATEN gains better performance over TSN in majority of action categories (Figure 6). Consequently, the total average accuracy is higher than TSN.

### 5.4. Evaluation on UCF101

**Figure 7:**
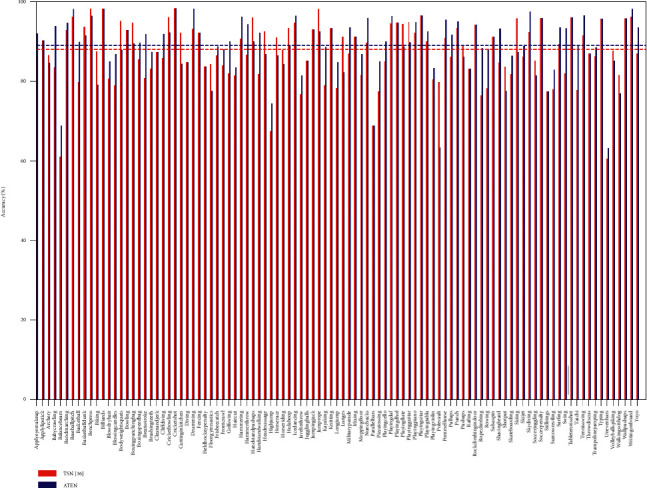
Performance comparison between ATEN and TSN (two modalities) [34] on UCF101.

**Figure 8:**
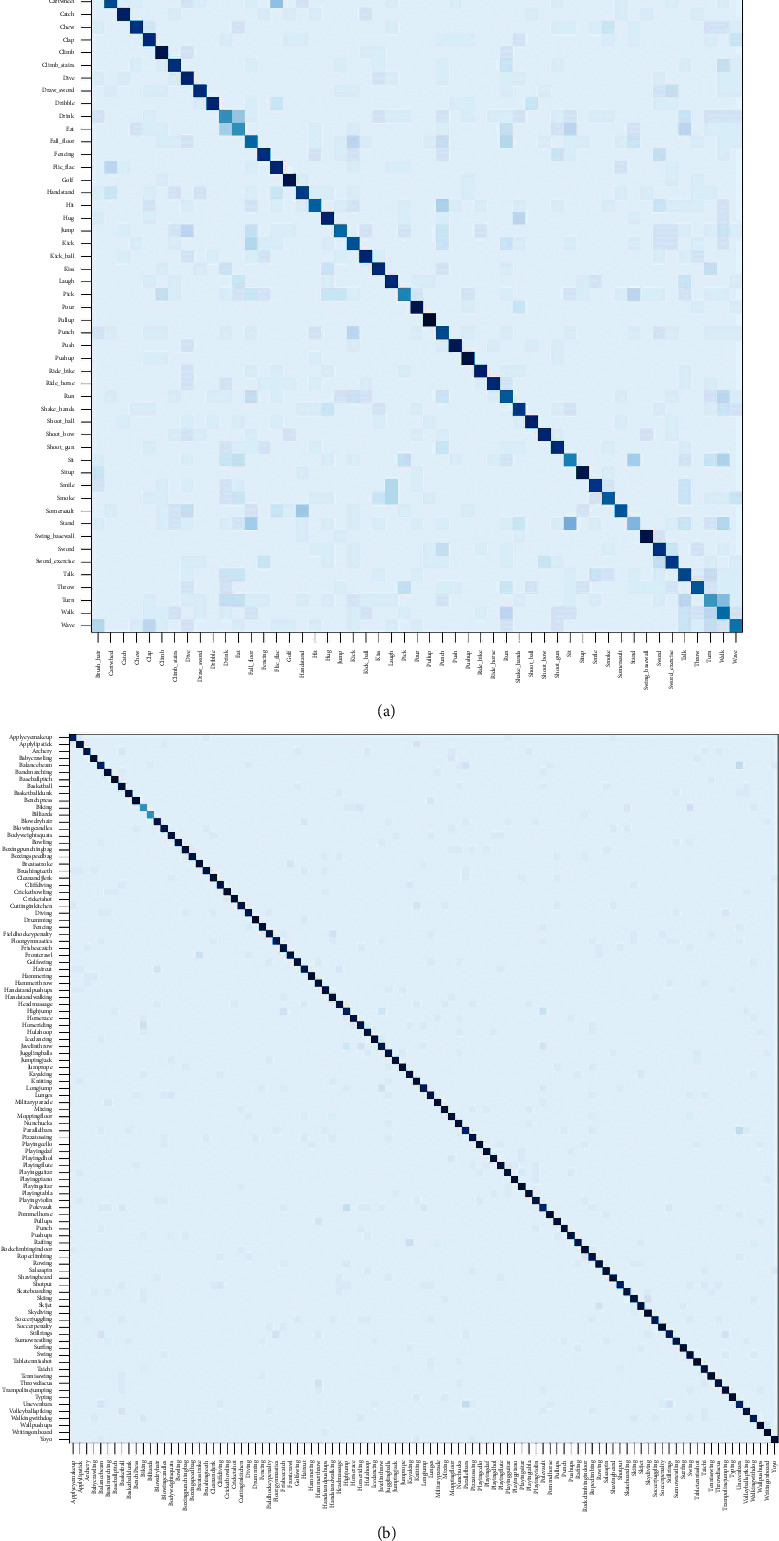
Performance confusion matrix on (a) HMDB51 and (b) UCF101 (two modalities).

As is given in Table 1, the recognition performance of ATEN on UCF101 with different modalities reaches 86.3%, 88.7%, and 94.5%, respectively. Interestingly, we observe that temporal stream in our framework achieves better performance on both two datasets compared with spatial stream and combining both of them leads to a higher accuracy, which is also consistent with [3, 27, 29]. To further investigate the performance of ATEN on UCF101, we report the individual category accuracy in Figure 7. Compared with TSN, the recognition accuracy on several typical actions such as “RopeClimbing,” “Swing,” and “TaiChi” is improved significantly. This improvement mainly comes from the use of the proposed framework. On one hand, the video features are extracted more concentrating from the action-related areas by BIMM, and on the other hand, the attention-based model in ATEN adaptively eliminates the temporal redundancy which makes the video representation more discriminative.  Figure 8 shows the performance confusion matrix on two datasets. We can find that some actions are easily confused into wrong categories especially when the two actions are similar, such as “Throw” and “Wave,” “Eat” and “Drink.” In spite of that, our method still obtains promising performance on these easy-confused actions as well as on most of the actions.

### 5.5. Ablation Study

Here, for the verification of the effectiveness exhibited by the proposed background-independent motion mask (BIMM) and attention-based temporal encoding network (ATEN), various ablation experiments were performed using a range of settings on 2 datasets: RGB + OF + average fusion (Setting 1, similar with TSN), RGB + OF + ATEN (Setting 2), RGB-BIMM + OF-BIMM + average fusion (Setting 3), and RGB-BIMM + OF-BIMM + ATEN (Setting 4). Tables 3 and 4 separately present the experimentally achieved results on 2 datasets. The best value in the table is presented in bold. According to the experimental results, Setting 4 (RGB-BIMM + OF-BIMM + ATEN) achieves the superior performance in 4 settings on both two datasets. From Tables 3 and 4, we can find that the temporal encoding module is more critical than BIMM on the UCF101 dataset. The accuracy of Setting 1 and Setting 3 is basically the same (93.0 vs 93.3). We speculate that this result is explained as the video background of the UCF101 dataset is relatively simple, so whether to use BIMM or not has little effect on recognition accuracy whereas on the HMDB51 dataset, both BIMM and temporal encoding modules provide strong contribution to the recognition accuracy. It is not hard to find out that without any of BIMM or temporal encoding module is likely to cause recognition accuracy to decline.

### 5.6. Comparison with the Existing

Finally, the performance of our work is compared with several existing methods on two datasets.  Table 5 lists the results. The optimal value in the table is presented in bold. The approach here is compared with not only handcraft approaches (e.g., improved dense trajectories (IDT) [12], saliency-based trajectory (ST) [25], and spatiotemporal motion skeleton representation [26]), but also deep learning representations (e.g., 3D-CNN (C3D) [11], two-stream CNN [27], trajectory-pooled deep-convolutional descriptors (TDD) [30], motion-salient-region convolutional neural network (MSR-CNN) [10], tube convolutional neural network (T-CNN) [32], sequential trajectory texture (STT) deep representation [33], three-stream CNN [29], long-term temporal convolutional network (LTC) [35], and temporal segment network (TSN) [34] with RGB and OF modalities). Moreover, the method here is compared with many attention-based methods such as dual attention convolutional network (DANet-50) [39], spatial-temporal attention network (STA-CNN) [42], spatiotemporal attention module (SAM) [41], and unified spatiotemporal attention networks (STANs) [40]. We present the final results of ATEN with 6 temporal segments and fuse the predicted data of RGB-BIMM and OF-BIMM modalities with the weights of 1 : 1.5. The accuracy reaches 70.6% on the HMDB51 dataset and 94.5% on the UCF101 dataset, which is rather competitive. Our final results outperform most of the methods averagely by 7.8% on the HMDB51 dataset and 4.0% on the UCF101 dataset, which demonstrates the effectiveness of BIMM in motion segmentation and the significance of ATEN in long-term temporal encoding in videos. Meanwhile, the STA-CNN model on HMDB51 has better result than here, and we conjecture that the major ConvNet of ATEN (i.e., BN-Inception) is different from the backbone architectures of STA-CNN. With a more novel architecture selected as the major ConvNet of ATEN, higher performance is likely to be achieved.

### 5.7. Visualization of BIMM and Learned Temporal Attention

**Figure 9:**
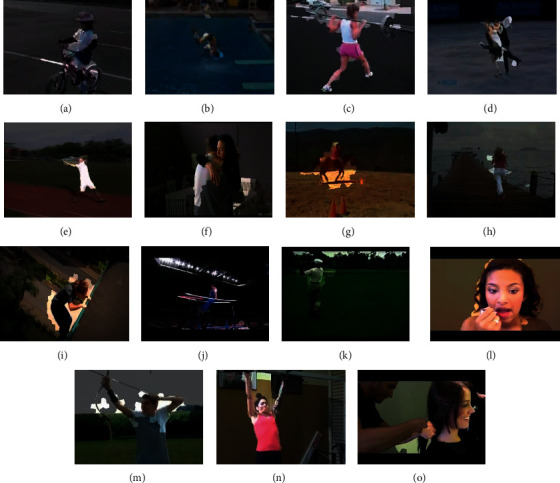
Visualization of motion segmenting approaches on the basis of boundary prior for BIMM on two datasets. (a) Biking, (b) Diving, (c) Lunges, (d) IceDancing, (e) JavelinThrow, (f) Hug, (g) Ride horse, (h) Run, (i) Jump, (j) ParallelBars, (k) Golf, (l) ApplyLipstick, (m) Archery, (n) PullUps, and (o) Haircut.

To verify the importance of BIMM for classification, Figure 9 presents the visualization of boundary prior-based motion segmentation for BIMM on two datasets, in which the respective sampled frame represents one action. We randomly select 15 actions from HMDB51 and UCF101 datasets in terms of visualizing process. To a certain extent, videos within the first two rows undergo the contamination with drastic background motion or other noises. The proposed BIMM is implemented on all sampled frames, from which the motion areas are brighter than the background. This also indicates that the region with more visual information is preserved, because the convolution focuses more on these areas. It is worth noting that the segmented foreground always seems larger than the ground truth in each frame. The reason is possibly that the BIMM is computed from the frame column and thus cover the motion areas pertaining to overall frames inside one time window instead of a single static frame (Figure 9). The frames in the third row show actions with a static background, in which the moving objects are also well segmented. These visualized frames demonstrate that the motion segmenting approaches on the basis of boundary prior perform well in most cases.

**Figure 10:**
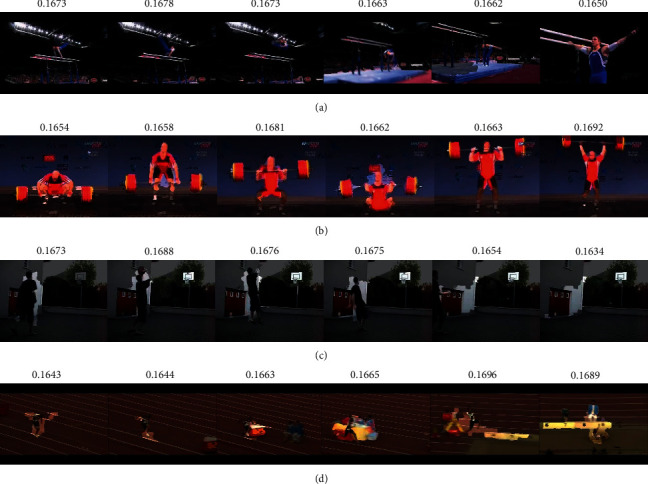
Illustration of the learned attentions on four actions over temporal dimension. (a) ParallelBars, (b) CleanAndJerk, (c) Basketball, and (d) LongJump.

To analyse the effects of the model of attention and to visualize the key snippets from long-term temporal segments proposed in ATEN, the temporal encoding results are visualized from video segments to reveal how the temporal encoding aggregation represents the target actions.  Figure 10 illustrates which input frames from the snippets to cause the attention *w*_att_ in the temporal domain. The proposed ATEN prioritizes discriminative video frames (the most critical stages) by assigning higher attention values to the specific actions. Naturally, less informative frames are assigned with lower attention weights as expected. As shown in Figure 10(d), frames within the athlete jumping upon the sandpit are assigned higher attention values than that of the athlete running on the playground because the frames that contain both athlete and sandpit are more discriminative for classifying the action “LongJump.”

### 5.8. Evaluation of Computational Complexity

For evaluating the computational complexity of the proposed ATEN, we compute the average time consumption per video for our action recognition framework, including preprocessing, CNN-based feature extraction, and feature aggregation on the HMDB51 dataset. The experiment compared the proposed ATEN with TSN in Keras with 16G RAM and GTX1080ti, and the codes do not receive the optimization. The time consumption for the above framework is compared in Table 6. The preprocessing of ATEN is somewhat more computationally expensive than TSN. It is because the BIMM is used to enhance the moving objects from complicated background in ATEN. However, the time consumption for CNN-based feature extraction is much smaller than TSN, and we conjecture that the background in RGB-BIMM and OF-BIMM is relatively simple compared with the original frame, so the feature extraction computation can more significantly save time. Furthermore, the time consumption is not determined by model training and optical flow since the process of the mentioned models is consistent.

## 6. Conclusion

Here, the attention-based temporal encoding network (ATEN) based on attention is presented with background-independent motion mask (BIMM) for human action recognition which effectively reduces the side effect from both spatial and temporal redundant information and noises. Various experimental processes on 2 thought-provoking datasets verified the superior performance exhibited by the action recognition framework here. It is largely ascribed to the temporal encoding for the sampled snippets, as well as the automatically foreground segmentation method designed here. The former can effectively and efficiently deal with the long-term dependency among frames, whereas the latter makes the convolution focus more on the action-related areas. In the future, we intend to explore the combination of ATEN with some recurrent models such as RNN or LSTM to further exploit the temporal structure in videos to recognize actions of humans.

## Figures and Tables

**Table 1:** Accuracy (%) of proposed ATEN on HMDB51 and UCF101 datasets with different modalities.

Models	Accuracy (%)	
	HMDB51	UCF101
RGB-BIMM	59.7	86.3
OF-BIMM	62.2	88.7
Two modalities	70.6	94.5

**Table 2:** Top-10 performance improvement of ATEN over TSN [34] on HMDB51 and UCF101.

HMDB51		UCF101	
No.	Action category	Increment (%)	Action category	Increment (%)
1	Run	15.71	RopeClimbing	5.88
2	Pullup	14.29	Swing	5.62
3	Dive	12.86	TaiChi	5.56
4	Draw_sword	11.43	BreastStroke	5.48
5	Eat	10.00	HandstandWalking	5.19
6	Handstand	9.99	BabyCrawling	5.15
7	Wave	9.65	Basketball	5.05
8	Brush_hair	8.57	Rowing	4.95
9	Drink	8.24	Kayaking	4.76
10	Clap	7.14	ShavingBeard	4.24

**Table 3:** Ablation study on UCF101 (%).

Settings	BIMM	ATEN	Accuracy
Setting 1	✗	✗	93.0
Setting 2	✗	✓	94.1
Setting 3	✓	✗	93.3
Setting 4	✓	✓	**94.5**

**Table 4:** Ablation study on HMDB51 (%).

Settings	BIMM	ATEN	Accuracy
Setting 1	✗	✗	67.6
Setting 2	✗	✓	68.2
Setting 3	✓	✗	69.5
Setting 4	✓	✓	**70.6**

**Table 5:** Comparison with existing on HMDB51 and UCF101.

HMDB51		UCF101	
Methods	Accuracy (%)	Methods	Accuracy (%)
IDT [12]	57.2	IDT [12]	85.9
ST [25]	58.9	C3D [11]	85.2
STMS [26]	58.2	Two-stream CNN [27]	88.0
Two-stream CNN [27]	59.4	TDD [30]	90.3
TDD [30]	63.2	MSR-CNN [10]	93.7
MSR-CNN [10]	66.9	T-CNN [32]	87.5
STT [33]	65.2	STT [33]	92.2
Three-stream CNN [29]	68.5	Three-stream CNN [29]	93.4
LTC [35]	64.8	LTC [35]	91.7
TSN (two modalities) [34]	68.5	TSN (two modalities) [34]	94.0
DANet-50 [39]	54.3	DANet-50 [39]	86.7
STA-CNN [42]	**71.5**	STA-CNN [42]	93.8
SAM [41]	69.1	SAM [41]	94.3
STAN [40]	—	STAN [40]	93.6
ATEN (two modalities)	70.6	ATEN (two modalities)	**94.5**

**Table 6:** Comparison of the time consumption on average of each video.

Framework	Preprocessing	CNN-based feature extraction	Feature aggregation	Total
TSN [34]	78.6	13.3	1.2	93.1
ATEN	103.2	9.1	1.1	113.4

## Data Availability

The experimental data used to support the findings of this study were supplied by Zhengkui Weng under license and so cannot be made freely available. Requests for access to these data should be made to the corresponding author.
